# Emphysematous Cholecystitis Discovered on a Chest Radiograph

**DOI:** 10.5334/jbr-btr.1130

**Published:** 2016-06-23

**Authors:** Patrick Ndja, Hanna Salame, Nigel Howarth, Denis Tack

**Affiliations:** 1 Epicura, BE; 2 Clinique des Grangettes, CH

**Keywords:** emphysematous cholecystitis, chest radiograph, computed tomography

## Clinical History

A 76-year-old man without any significant medical history except arterial hypertension presented in our emergency department during the night with complaints of right lower chest pain. Physical examination and pulmonary auscultation were unremarkable. There was no fever. Chest and right ribs radiographs were obtained and considered unremarkable for chest disease by the emergency physician, who considered the final diagnosis of an intercostal tear. It was treated with analgesics.

**Figure 1:**
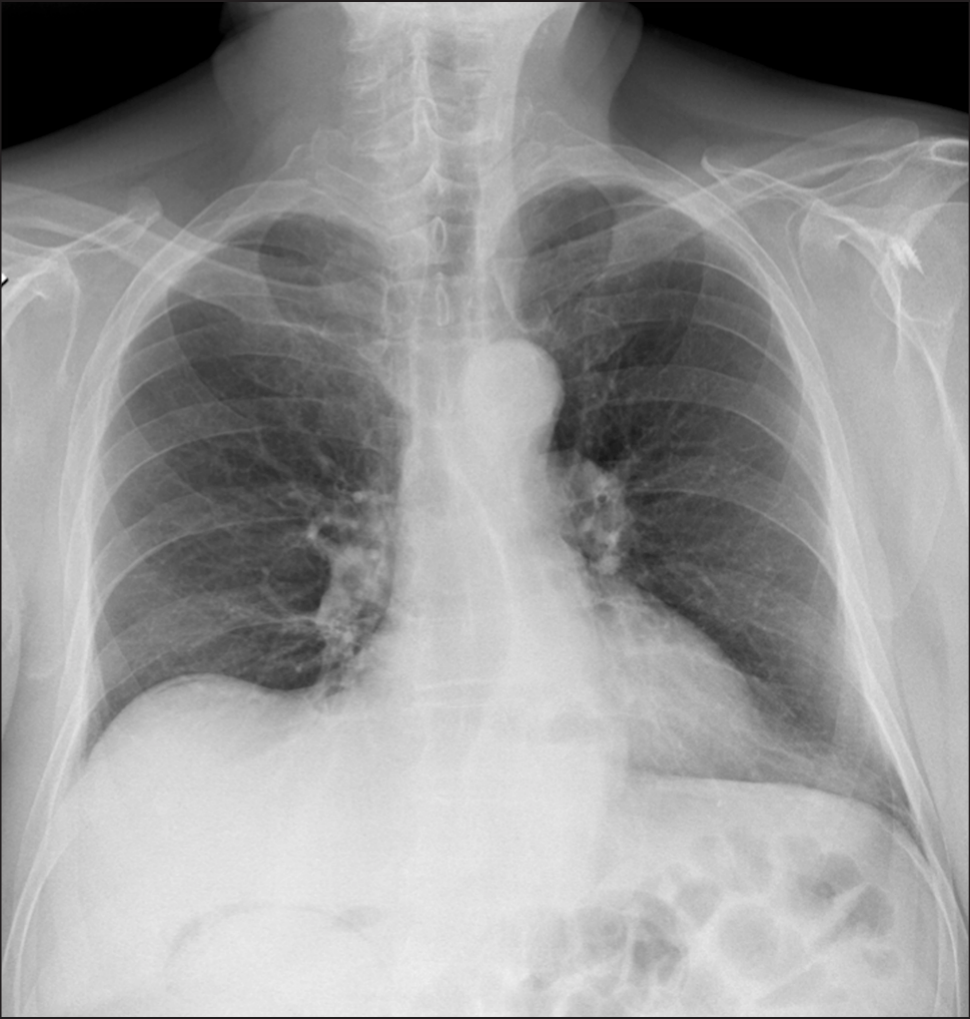


**Figure 2:**
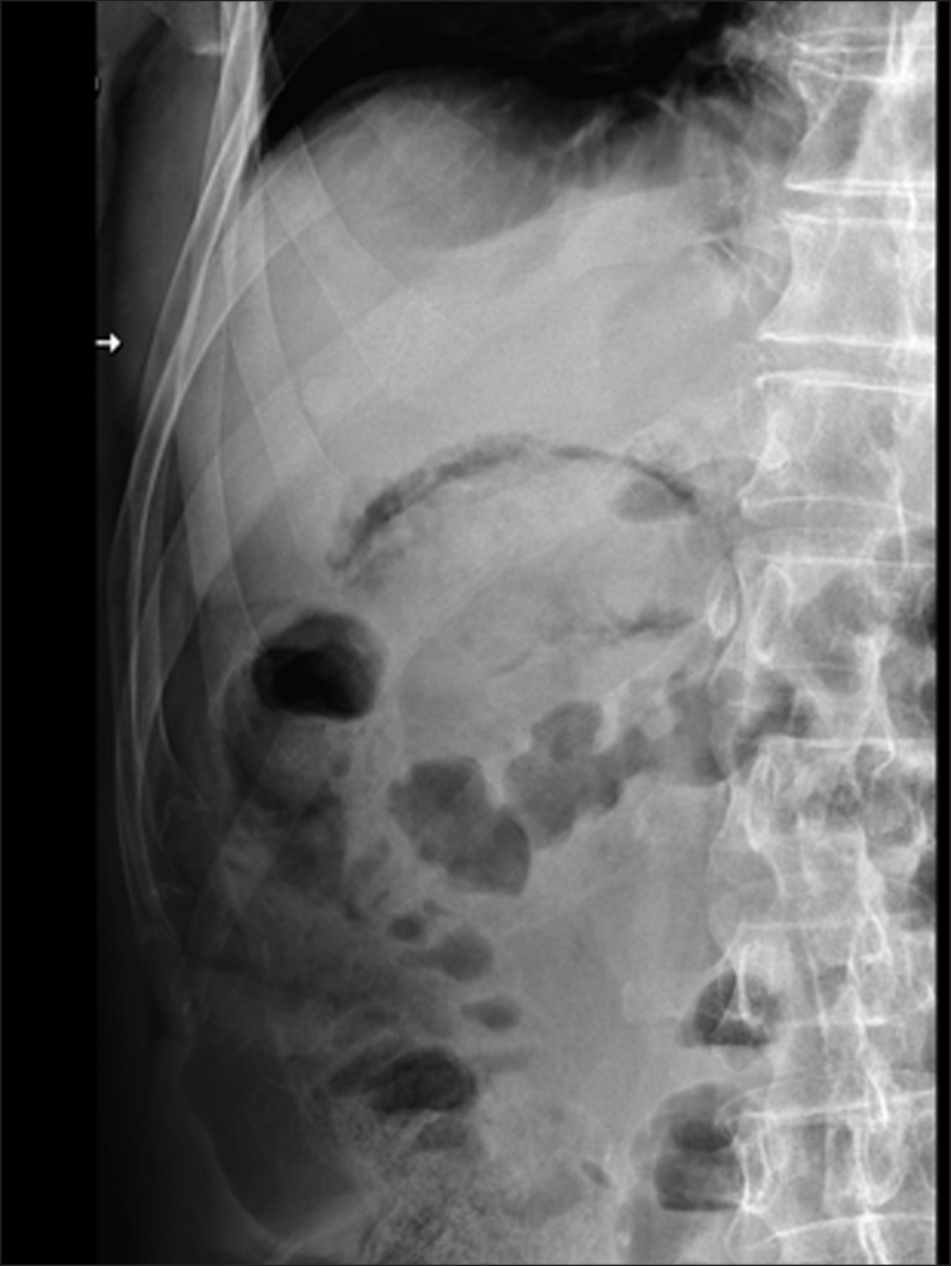


**Figure 3:**
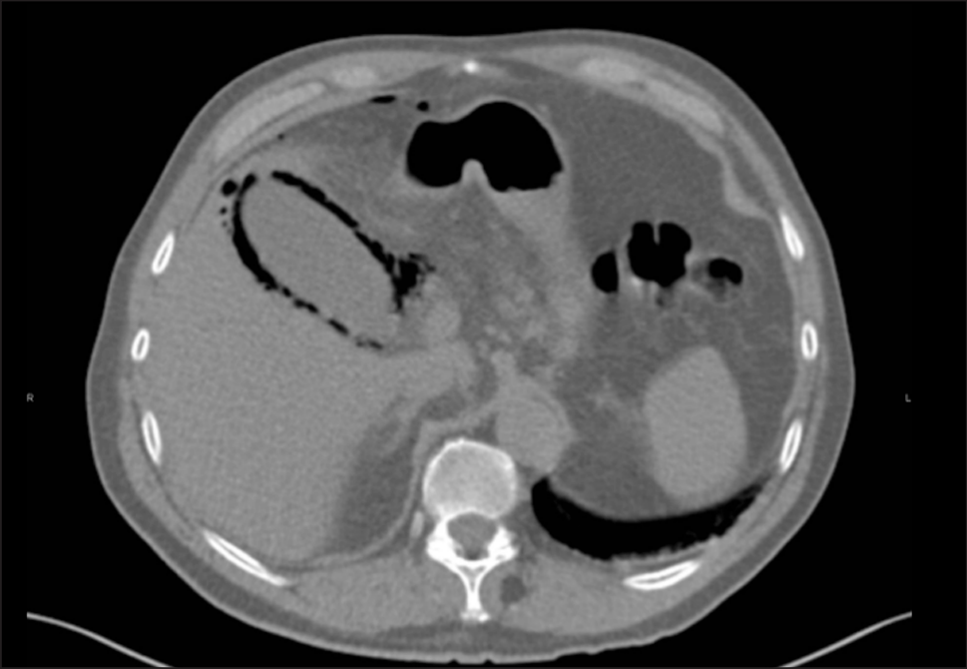


During its review of the plain films the next morning, the attending radiologist found a small air collection below the right hemidiaphragm and a gaseous halo below the liver area (Figures 1 and 2). The patient was called back, and an unenhanced abdominal CT was performed. The CT confirmed the pneumoperitoneum and precisely located the gaseous halo in the wall of the gallbladder (Figure 3). A laparoscopic cholecystectomy was performed the same day. Pathological analysis of the surgical specimen showed a necrotic cholecystitis with abscesses but without any malignancy.

## Discussion

Emphysematous cholecystitis is also called “pneumocholecystitis” [1]. This is a rare and severe form of acute cholecystitis in which the arterial ischemic process is complicated by an anaerobic bacterial superinfection producing gas in the gallbladder wall. A male preponderance is noted, diabetes is present in over 50 per cent, and a third of patients do not carry any calculus. The clinical picture is often little alarming, with apyrexia in one-third of cases, the pain being dwindled by diabetic neuropathy. Biologically, there is some increase in white blood cell count, mainly through neutrophils associated with inflammation [1]. White blood cell count may be normal in up to one-third of the cases.

The prognosis can be poor, depending on co-morbidities, with mortality rising up to 15 per cent whereas it is below 4.1 per cent in usual acute cholecystitis. Pneumoperitoneum occurs rarely but has a poor prognosis due to the spread of the sepsis in the entire abdominal cavity. CT is the most sensitive technique for the diagnosis of emphysematous cholecystitis. It allows affirming the presence of air in the gallbladder wall or lumen and in the surrounding tissues, requiring prompt surgical management.
